# Brain and serum metabolomic studies reveal therapeutic effects of san hua decoction in rats with ischemic stroke

**DOI:** 10.3389/fendo.2023.1289558

**Published:** 2023-11-30

**Authors:** Ruisi Liu, Shengxuan Cao, Yufeng Cai, Mingmei Zhou, Xiaojun Gou, Ying Huang

**Affiliations:** ^1^ Institute of Interdisciplinary Integrative Medicine Research, Shanghai University of Traditional Chinese Medicine, Shanghai, China; ^2^ Experimental Research Center, China Academy of Chinese Medical Sciences, Beijing Key Laboratory of Research of Chinese Medicine on Prevention and Treatment for Major Diseases, Beijing, China; ^3^ Shanghai Frontiers Science Center of TCM Chemical Biology, Shanghai University of Traditional Chinese Medicine, Shanghai, China; ^4^ Central Laboratory, Baoshan District Hospital of Integrated Traditional Chinese and Western Medicine of Shanghai, Shanghai University of Traditional Chinese Medicine, Shanghai, China

**Keywords:** Chinese medicine formula, ischemic stroke, inflammation, metabolomics, GC-MS

## Abstract

San Hua Decoction (SHD) is a traditional four-herbal formula that has long been used to treat stroke. Our study used a traditional pharmacodynamic approach combined with systematic and untargeted metabolomics analyses to further investigate the therapeutic effects and potential mechanisms of SHD on ischemic stroke (IS). Male Sprague-Dawley rats were randomly divided into control, sham-operated, middle cerebral artery occlusion reperfusion (MCAO/R) model and SHD groups. The SHD group was provided with SHD (7.2 g/kg, i.g.) and the other three groups were provided with equal amounts of purified water once a day in the morning for 10 consecutive days. Our results showed that cerebral infarct volumes were reduced in the SHD group compared with the model group. Besides, SHD enhanced the activity of SOD and decreased MDA level in MCAO/R rats. Meanwhile, SHD could ameliorate pathological abnormalities by reducing neuronal damage, improving the structure of damaged neurons and reducing inflammatory cell infiltration. Metabolomic analysis of brain and serum samples with GC ***-*** MS techniques revealed 55 differential metabolites between the sham and model groups. Among them, the levels of 12 metabolites were restored after treatment with SHD. Metabolic pathway analysis showed that SHD improved the levels of 12 metabolites related to amino acid metabolism and carbohydrate metabolism, 9 of which were significantly associated with disease. SHD attenuated brain inflammation after ischemia-reperfusion. The mechanisms underlying the therapeutic effects of SHD in MCAO/R rats are related to amino acid and carbohydrate metabolism.

## 1 Introduction

Stroke is recognized as one of the most serious health and life-threatening diseases. Although the incidence of stroke has stabilized and mortality has decreased in recent years, the global burden of stroke continues to grow (1). Strokes are generally classified as hemorrhagic or ischemic stroke (IS), with the latter accounting for approximately 85% of all stroke types (2). IS is severe brain tissue necrosis due to stenosis or obstruction of the arteries supplying blood to the brain (carotid and vertebral arteries), as well as inadequate blood supply to the brain. Its disability rate and mortality rate are also much higher than other types (3).

The clinical treatment options for stroke are very limited and the time window for treatment is also quite narrow. Many contraindications place limitations on the treatment of stroke (4). Tissue-type plasminogen activator (tPA) is currently the only thrombolytic agent approved by the U.S. Food and Drug Administration (FDA) for the treatment of IS (5). However, delayed thrombolytic therapy with tPA has great potential to increase this aspect of hemorrhagic transformation. Thus, there is a pressing need to find better treatment options to overcome the limitations and adverse effects of clinical treatment.

Traditional Chinese medicine (TCM) has been widely used in the treatment of stroke due to its rich application experience and unique theoretical system (6, 7). TCM and has significant advantages in terms of safety, efficacy and multi-target profiling in the treatment of IS (8). According to the development level of IS, different herbal formulas can be selected for treatment (9). It has been shown that some bioactive components in Chinese herbs promote endogenous neurogenesis by affecting the proliferation, migration and differentiation of neural stem cells after reperfusion, which has great therapeutic effects in improving neurological function and cerebral infarct area (6). Salvianolic acid A administration significantly reduced infarct volume and vascular embolization in MCAO/R rats, ameliorated pathological damage in the hippocampus and striatum as well as neurological deficits (10). Ginseng Shouwu extract improved learning and memory abilities in vascular dementia rats. It also promoted the increase in the number of newborn neurons and brain microvessel density by inhibiting the TLR4/NF-κB/NLRP3 inflammatory signaling pathway after IS in rats (11). Baimi Decoction improved neuronal function and neurogenesis by decreasing neuronal loss, vacuolization, neuronal atrophy and neuronal structural disruption, and modulation of the expression of vascular endothelial growth factor, caspase-3 and NF-κB to alleviate pathological abnormalities in MCAO/R rats (12). In addition, Chinese herbal medicine has been shown to reduce the side effects of tPA thrombolysis for acute IS as an adjunctive therapy (13).

San Hua Decoction (SHD) is a symbolic traditional Chinese herbal fomula for IS, first included in “Suwen Bingji Qiyi Baomingji” (a traditional Chinese medicine classic of 1188 A.D. in Jin Dynasty). Clinically, SHD ameliorates hemiplegia due to stroke and stroke sequelae. It has been shown that SHD can effectively improve the prognosis and blood rheology of patients with acute stroke by regulating phosphorylated tau levels, promoting endogenous neurogenesis and reducing cerebral infarction after ischemia-reperfusion injury, and facilitating patient recovery (14). In addition, SHD also has a preventive effect on IS and can be used to improve cerebral embolism and hypertensive crisis (15). SHD contains four herbs, Rhei Radix et Rhizoma (*Rheum palmatum* L. [Polygonaceae], Dahuang) (RR), Notopterygii Rhizoma et Radix (*Notopterygium incisum* Ting ex H. T. [Apiaceae], ChangQianghuo) (NR), Magnoliae Officinalis Cortex (*Magnolia officinalis* Rehd.et Wils. [Magnoliaceae], Houpu) (MO), and Aurantii Fructus Immaturus (*Citrus aurantium* L. [Rutaceae], Zhishi) (AF). Modern pharmacological studies have found that all four herbs have effects related to IS therapy. RR inhibits platelet aggregation and protects neurons damaged from hypoxic-ischemic brain injury (16). The anthraquinone constituent of RR has a protective effect against IS (17). NR can increase cerebral blood flow and improve cerebral blood circulation to reduce neuropathic pain (18). MO can protect nerves from ischemic and reperfusion injury through suppression of encephalitis and improvement of BBB dysfunction. It has preventive and therapeutic effects on neurological and psychiatric diseases (19, 20). The peel extract of AF has been shown to have anti-inflammatory, antioxidant and anti-apoptotic effects through AMPK and NRF2-related signaling, alleviating liver damage in mice (21). The potential therapeutic mechanisms of SHD for IS remain unclear, but we hypothesize that SHD can influence disease progression by improving metabolism.

In this research, we evaluated the therapeutic effects of SHD in a rat model of middle cerebral artery occlusion reperfusion (MCAO/R) with conventional pharmacodynamic indicators, combining a metabolomics approach to identify differential metabolites in brain and serum to elucidate the potential mechanisms of SHD on IS.

## 2 Materials and methods

### 2.1 Materials and reagents

Methanol, methoxamine and N, O-Bis (trimethylsilyl) trifluoro-acetamide (BSTFA with 1% TMCS) were provided by Sigma-Aldrich Co., Ltd. (Germany); chloroform and pyridine were obtained from Sinopharm Chemical Reagent Co., Ltd (Shanghai, China); heptadecanoic acid was purchased from Aladdin Reagent Co., Ltd. (Shanghai, China). All reagents are analytical grade.

### 2.2 Preparation of san hua decoction

SHD consists of 4 traditional Chinese herbs, namely Rhei Radix et Rhizoma (RR), Notopterygii Rhizoma et Radix (NR), Magnoliae Officinalis Cortex (MO) and Aurantii Fructus Immaturus (AF). The medicinal parts of RR, NR, MO and AF were the dried roots, dried barks (including root and branch barks), and dried fruits of the original herbs, respectively. All Chinese medicine decoction slices were purchased from Beijing Tongrentang Co., Ltd. (Beijing, China) and authenticated by Dr. Xirong He (China Academy of Chinese Medical Sciences). The herbs used in SHD were tested for the detection of microorganisms, heavy metals and pesticide residues before they were sold. All test results are in accordance with Chinese safety standards. The quality identification standards of the herbs used were in compliance with the 2020 edition Chinese Pharmacopoeia.

In preparation of SHD, the crude herbs (RR, NR, MO and AF) were mixed in a ratio of 1:1:1:1. The 4 herbs were ground into powder and mixed well to obtain 80 g of raw herb powder, then boiled in 10 times volume of sterile water for 30 minutes. The herbs were then decocted for 2 h, filtered and concentrated to obtain water-based decoctions containing 1 g/mL of the raw herbs. In our previous study, we used an HPLC method to analyze the main components of SHD. SHD mainly contains six active ingredients for the treatment of ischemic stroke, namely Rhein, Emodin, Chrysophanol, Neohesperidin, Magnolol and Notopterol (22).

### 2.3 Experimental animals

Male Sprague-Dawley rats (SD rats, 8 weeks, body weight 230-250 g) were obtained from Beijing Huafukang Biotechnology Co., Ltd (Beijing, China, SCXK (JING) 2019-0008). All animal experiments complied with the standards of the Principles for Laboratory Animals and were conducted under the guidance of the Bioethics Committee of Experimental Research center of China Academy of Chinese Medical Sciences (license number: ERCCACMS21-2307-03). The rats were housed in an environment with the temperature of 23–25°C and the humidity of 50% with 12 h day and night cycle. The rats were kept for 3 days before performing experiments. Normal rodent chow and water are available to all rats without restriction.

### 2.4 Grouping and drug administration

The 32 rats were grouped at random into control (Con), sham-operated (Sham), MCAO/R model (Model), and SHD groups, with 8 rats in each group by using the randomized numerical table method. The Con group received no treatment. Sham group received sham operation, Model group and SHD group received MCAO/R treatment. According to the weight of rats, the SHD group was treated with SHD by intragastric administration. The dose administered to rats in the SHD group was converted from the patient’s daily clinical dose, which was 7.2 g/kg administered at 10 a.m. daily. The Con, Sham and Model groups were given equal volumes of purified water in same manner, once a day for 10 days. The administration in all groups was uniformly done at the end of modeling and was a therapeutic intervention.

### 2.5 MCAO/R model establishment

The MCAO rat model was set up by the suture-occluded method according to our previous methods (23). Rats were anesthetized with 1% pentobarbital (40 mg/kg BW, i.p.). The right common carotid artery (CCA), internal carotid artery (ICA) and external carotid artery (ECA) of rats were exposed and carefully isolated without stimulating the vagus nerve. After ligating the CCA and ECA with a thin wire 5 mm from the bifurcation of the ECA and ICA and at the end of the CCA, respectively, the ICA blood flow is blocked by pulling the preloaded wire. A small incision was made at the bifurcation of ICA and CCA, and the monofilament was inserted. Loosened the thread and slowly advanced approximately 18–20 mm until resistance occurred. The ICA is then ligated, leaving the thread in place to prevent the monofilament from falling out. After 90 minutes of cerebral ischemia, the monofilament was gently removed from the ICA to perform reperfusion. Different from the Model group, the monofilament in the Sham group was only inserted 10 mm in the ICA, not the MCA. The wound was disinfected with iodine and sutured.

### 2.6 Cerebral infarct area measurement and histopathological examination

Rats were anaesthetized with 1% pentobarbital (40 mg/kg, *i.p*.) by intraperitoneal injection, 24 hours after the last administration of the drug. The brain was isolated after craniotomy. MCAO/R-induced cerebral infarct areas were confirmed by 2,3,5-triphenyltetrazolium chloride (TTC) staining. The brain tissue was cut into six posterior coronal slices before incubation with 2% TTC. The cerebral infarct area percentage was calculated by using the Image J 1.41 software (24). For histopathological examination, brain tissues from the ischemic area were fixed in 4% paraformaldehyde for 12 hours. The brain tissues was then subjected to gradient dehydration and transparency with xylene for 1 hour. Finally, the brain tissues were embedded in paraffin, sectioned and stained with hematoxylin and eosin (H&E).

### 2.7 Serum biochemical examination

The levels of superoxide dismutase (SOD) and malondialdehyde (MDA), two oxidative stress-related indicators, were measured using the appropriate assy kits.

### 2.8 Metabolomic studies

#### 2.8.1 Sample collection and procession

Metabolite profiles were analyzed using gas chromatography-mass spectrometry (GC-MS). 24 hours after the last dose, 1% pentobarbital (40 mg/kg *i.p.*) was used to anesthetize rats and blood was obtained from the abdominal aorta. Serum samples were obtained by centrifugation at 4°C, 3500 rpm for 15 minutes, and the supernatant was gathered. Brain tissue was put into normal saline (NS) to remove blood. The collected serum and brain samples were then frozen at -80°C. Subsequent analyses were performed by GC-MS within 48 hours.

#### 2.8.2 Sample preparation for GC-MS

Samples were thawed at room temperature before processing. The 50 mg of brain tissue was taken and 500 µl of methanol-water-chloroform (5:2:2, v/v/v) mixture was added. The mixture was vortexed for 1 minute and then sonicated for 5 min. The 50 μL of serum was placed in a 1.5 mL centrifuge tube. Centrifuge tubes were placed at -20°C and incubated for 20 minutes. After being centrifuged at 13,000 rpm for 10 min at 4°C, the supernatant was removed. The 10uL of heptadecanoic acid-methanol solution (1.0 mg/mL) was added into 200 μL of supernatant as internal standard. After being blow-dried, 50 μL of methoxyamine-pyridine hydrochloride solution (15 mg/mL) was added to the residue and shaken at 30°C for 90 min. After adding 50 μL of methoxylamine-pyridine hydrochloride solution (15 mg/mL) to the residue, it was shaken at 30°C for 90 min. The mixture was then methylsilylated at 70°C for 1 h after adding 50 μL of BSTFA with 1% TMCS to the mixture. The samples were left at room temperature for 1h and waited for analysis.

#### 2.8.3 GC-MS conditions

The Agilent 6890N gas chromatograph with the Agilent 5975B mass selective detector and an inert electron impact ionization (EI) source comprise the GC-MS analysis system. The column was an Agilent J&W DB-5ms Ultra Inert chromatography column (30m x 0.25mm, 0.25µm). The carrier gas consisting of high purity helium (99.9996%) was delivered at a constant flow rate of 1.0 mL/min. Split injection was performed with the split ratio of 2:1 and injection volume of 1.0mL. The temperature of both the mass spectrometry interface and the injection port is 260°C. The temperatures of the ion source and the quadrupole were 230°C and 150°C, respectively. The ionization voltage is 70 eV. The data was acquired in full scan mode with a scanning range of 50-500 m/z. A randomized cross-feeding order was used for all samples.

The brain is analyzed under the following conditions: an starting temperature of the GC was set at 90°C for 1 minute. The temperature is increased to 180°C at a rate of 10°C/min, then to 240°C at a rate of 5°C/min. The final temperature is raised to 290°C at 25°C/min for 11 minutes. The solvent delay time was 5 minutes.

The serum is analyzed under the following conditions: an starting temperature of the GC was set at 80°C for 2 minute. The temperature is increased to 240°C at a rate of 5°C/min, then to 290°C at a rate of 25°C/min for 10 minutes. The solvent delay time was 7 minutes.

#### 2.8.4 Data processing and pattern recognition analysis

The pre-processing of raw data was performed using R software. The data were further analyzed using SIMCA software, including orthogonal partial least squares discriminant analysis (OPLS-DA), S-plot and permutation validation. Metabolite changes between the two groups were analyzed using OPLS-DA. Permutation validation was performed prior to the OPLS-DA analysis. The parameters R2 and Q2 were used to assess whether the model was valid, so as to avoid the risk of overfitting. S-plots were obtained on the basis of variable importance in projection (VIP) values calculated by OPLS-DA with VIP values > 1.0.

### 2.9 Statistical analysis

All of the experimental measurements were presented as mean ± SD. Differential metabolite finding was analyzed by SPSS software for independent samples t-test. Determination of differential metabolites depended on VIP values > 1.0 and *p*-values < 0.05 from Student’s t-tests. GraphPad Prism software was used to analyze oxidative stress levels and differential metabolite levels. Shapiro–Wilk normality test to verify normality. One-way ANOVA was used for analyzing the statistics of four groups. The *post hoc* test used was the Dunnett-t test. Correlations between different metabolite levels and disease were determined by Spearman’s correlation analysis. The significant differences were indicated by: * *p* < 0.05, ** *p* < 0.01, *** *p* < 0.001.

### 2.10 Pathway analysis of metabolites

The different patterns of specific metabolites obtained by screening were analyzed using MetaboAnalyst 5.0 to identify their metabolic pathways. In this study, pathways with pathway impact >0.10 were categorized as potential metabolic pathways. All metabolic pathways were linked through the Kyoto Encyclopedia of Genes and Genomes (KEGG, http://www.genome.jp/kegg/).

## 3 Results

### 3.1 The influence of SHD on cerebral infarct area in rats with cerebral ischemia-reperfusion

The cerebral infarct area is an important indicator of brain damage. Compared with the Sham group, the cerebral infarct area of brain tissue in rats in the Model group was drastically higher, with statistical differences (*p* < 0.01). Compared with the Model group, the cerebral infarct area in the SHD group was significantly lower, with statistical differences (*p* < 0.01). Detailed data can be found in **Supplementary Figure 1**, **Supplementary Table 1**.

### 3.2 The influence of SHD on oxidative stress levels

**Figure 1:**
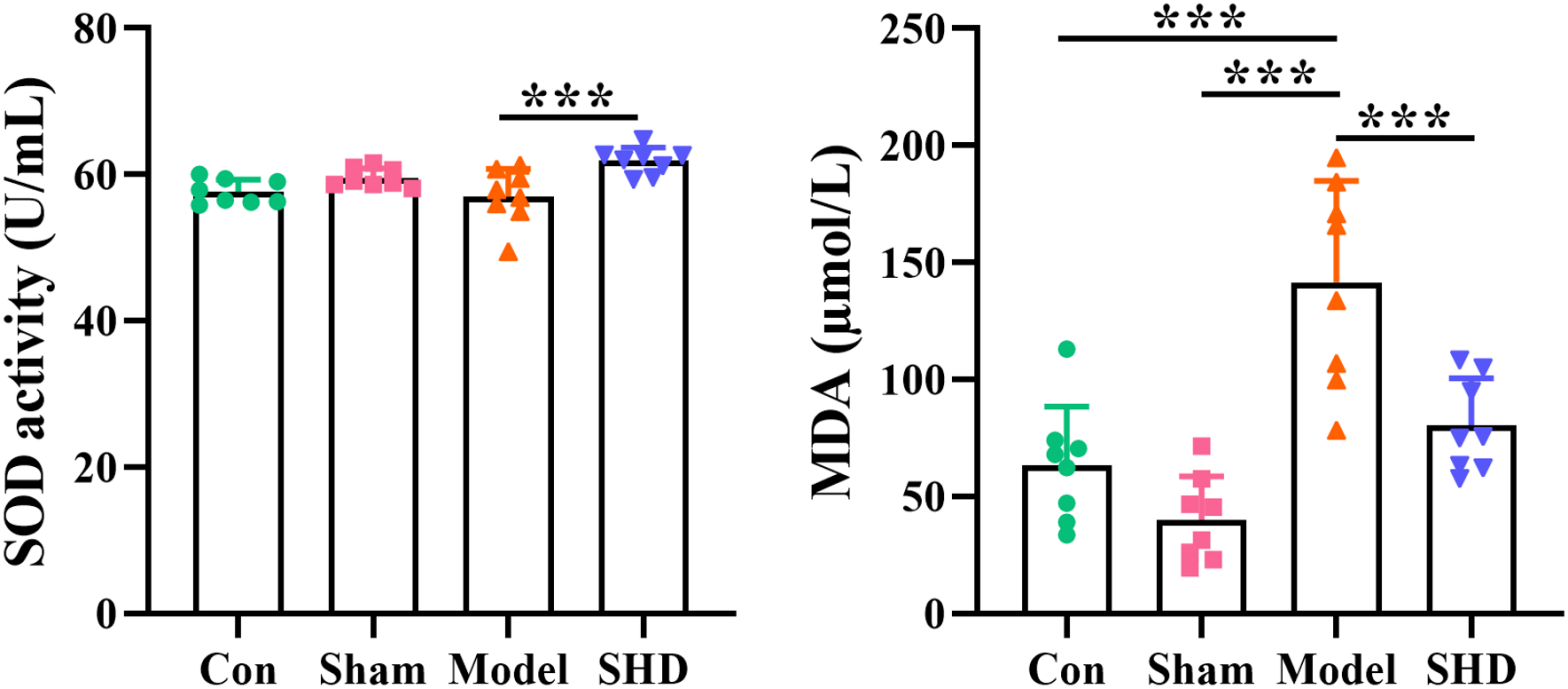
Effect of SHD on SOD and MDA in serum of rats with cerebral ischemia-reperfusion. ***p < 0.001 relative to the Model group.

The results of SOD and MDA measurements showed a beneficial role of SHD in regulating oxidative stress markers. Compared with the Sham group, MDA levels were significantly higher (*p* < 0.01) and SOD levels were not significantly changed in the Model group, while SOD was significantly higher in the SHD group (*p* < 0.01). Compared with the Model group, the SHD group showed an increase in SOD levels (*p* < 0.05) and a significant decrease in MDA levels (*p* < 0.01). (**Figure 1**, **Supplementary Table 2**).

### 3.3 The influence of SHD on the histopathological abnormality of brain

**Figure 2:**
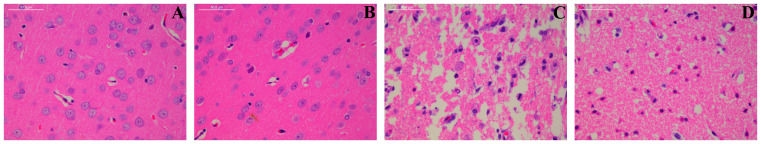
Effect of BMD on histopathological abnormality of brain tissue. Original magnification: ×4 and ×10. **(A)** Con group; **(B)** Sham group; **(C)** Model group; and **(D)** SHD group.

After H&E staining, the morphological characteristics of neurons were evaluated. Brain tissue morphology in the Con and Sham groups was essentially normal, with intact cytoarchitecture and neatly arranged cells, and no pathological abnormalities were observed (**Figures 2A, B**). In the Model group, the brain tissue in the ischemic area was extensively necrotic, with some cortical areas showing a highly sparse sieve reticular structure, unclear cell structure, significantly reduced number of brain tissue cells, and degeneration and necrosis of neurons (**Figure 2C**). The SHD group showed less cell necrosis and lesions, neater cell alignment, and less inflammatory cell infiltration compared to the Model group. (**Figure 2D**).

### 3.4 The influence of SHD on differential metabolites

#### 3.4.1 Multivariate data analysis for brain and serum samples

**Figure 3:**
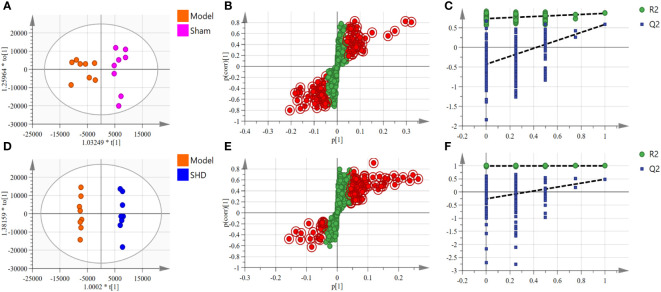
OPLS-DA scores, S-plot and permutation validation of the brain samples. **(A–C)** Sham group vs. Model group; **(D–F)** Model group vs. SHD group. (n = 8).

**Figure 4:**
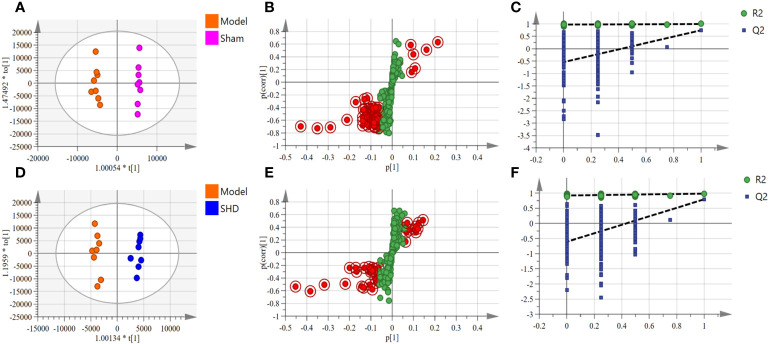
OPLS-DA scores, S-plot and permutation validation of the serum samples. **(A–C)** Sham group vs. Model group; **(D–F)** Model group vs. SHD group. (n = 8).

We used OPLS-DA, S-plot scores and permutation validation in supervised mode to analyze data from Sham, Model and SHD groups (**Figures 3**, **4**) to investigate the effect of SHD on the metabolic pattern of MCAO/R rats. A clear separation was observed between the Sham and Model groups (**Figure 3A**), indicating that the composition of brain tissue metabolites was significantly influenced by the MCAO/R procedure. The samples in the SHD group were significantly separated from the Model group (**Figure 3B**). The separation between the two groups indicates that the metabolites in the two groups are significantly different. This result confirms the effect of SHD treatment in ameliorating brain injury by modulating certain metabolic pathways.

#### 3.4.2 Identification of potential endogenous metabolites

**Figure 5:**
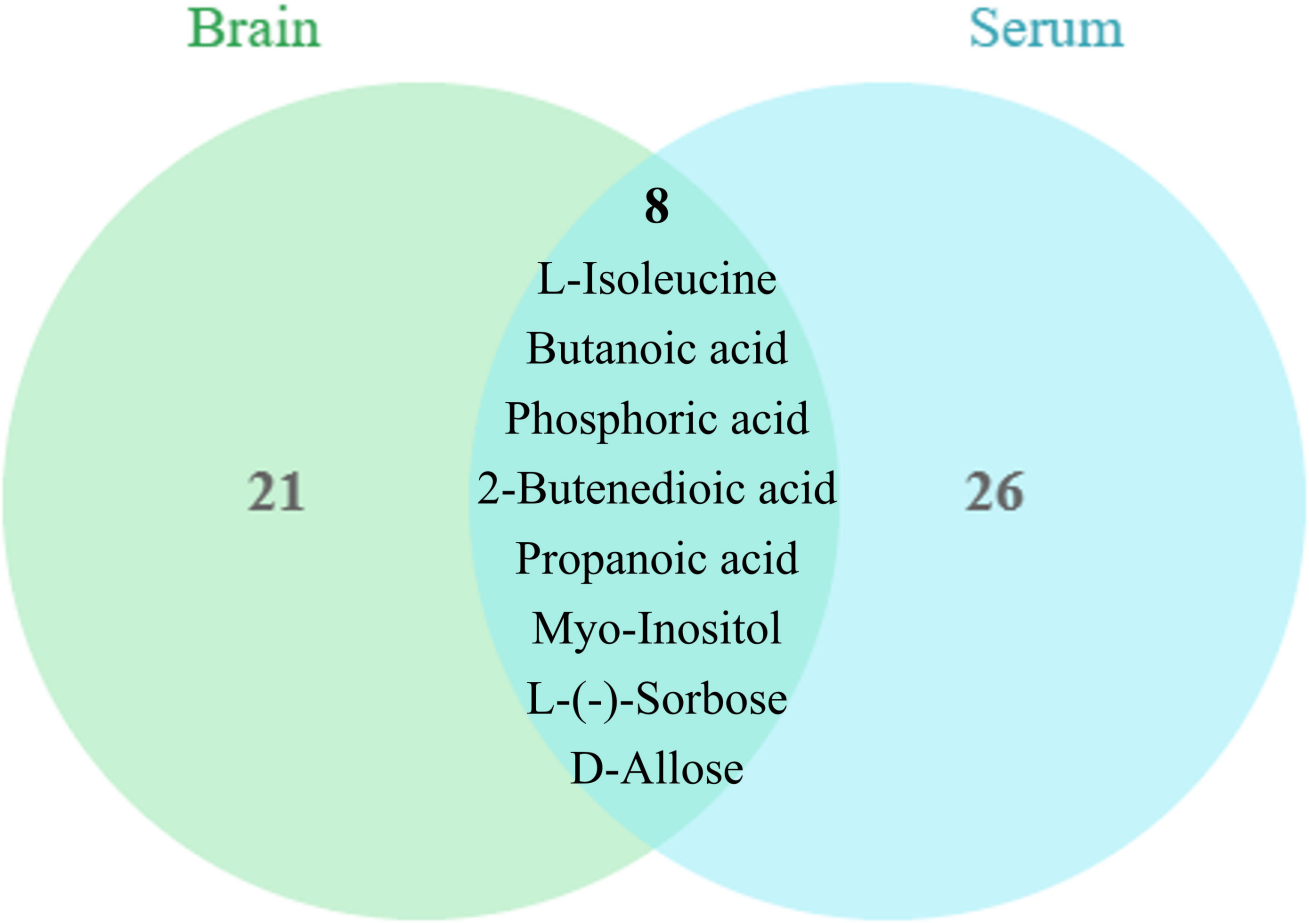
Metabolite differences between brain and serum samples. The brain and serum have 9 common differential metabolites, phosphoric acid, L-isoleucine, 2-butenedioic acid, propanoic acid, butanoic acid, L-(-)-sorbose, D-allose, D-Glucose and myo-inositol.

**Figure 6:**
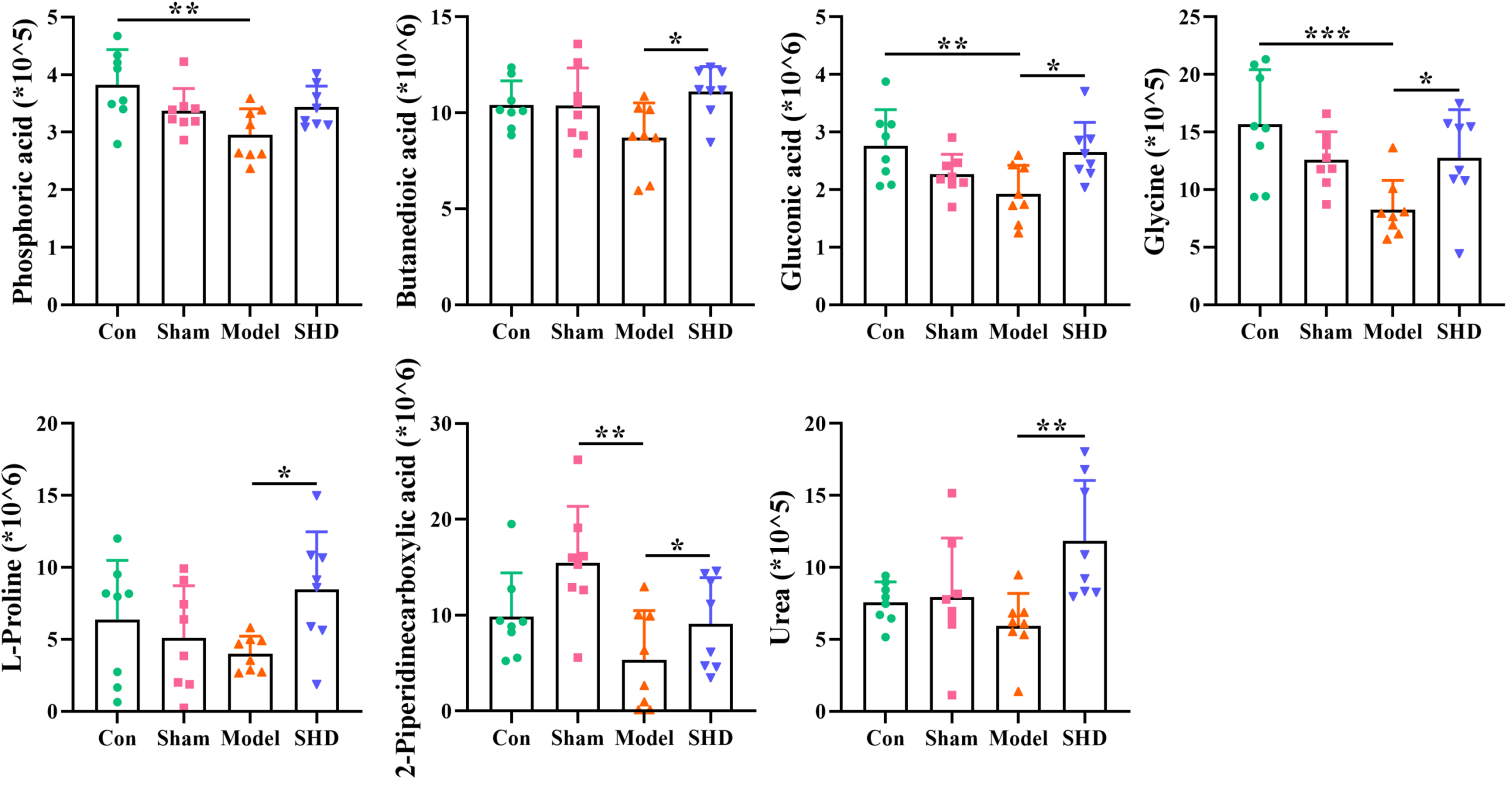
Differential metabolite changes in Model with SHD intervention in the brain. All values were presented as the mean ± SD. *p < 0.05, **p < 0.01, ***p < 0.001 relative to the Model group.

**Figure 7:**
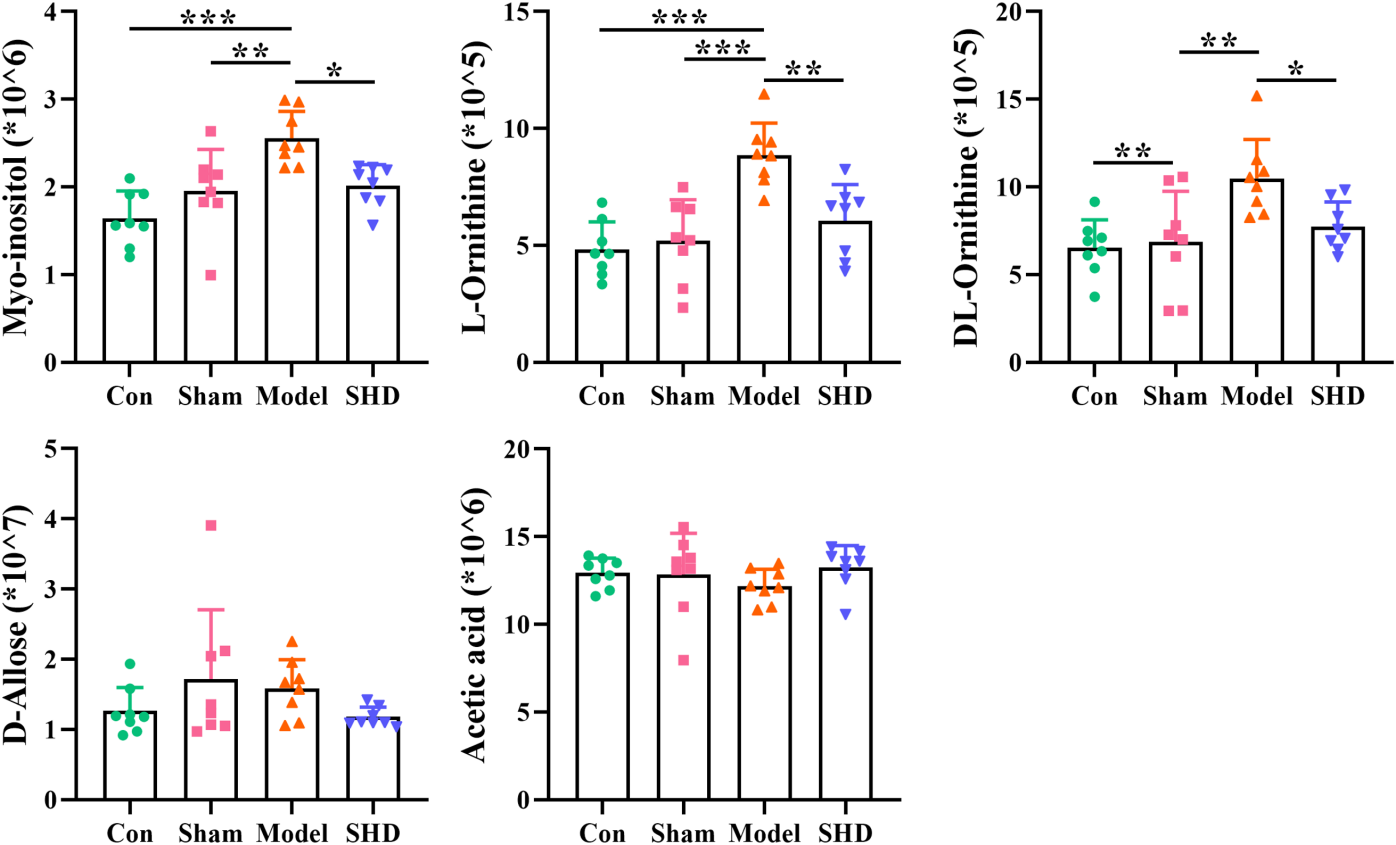
Differential metabolite changes in Model with SHD intervention in the serum. All values were presented as the mean ± SD. *p < 0.05, **p < 0.01, ***p < 0.001 relative to the Model group.

Screening for differential metabolites between the two groups associated with IS was performed by the OPLS-DA model. Significance levels were set at *p* < 0.05 and VIP > 1. We identified differential metabolites in the model and Sham groups. There were 28 different endogenous metabolites identified in the brain samples (**Table 1**). There were 34 different endogenous metabolites identified in the serum samples (**Table 2**). The overlap of endogenous metabolites in brain and serum samples was analyzed using Venn diagrams (**Figure 5**). The results showed that the following metabolites were present in serum and brain samples, phosphoric acid, L-isoleucine, 2-butenedioic acid, propanoic acid, butanoic acid, L- (–)-sorbose, D-allose,D-Glucose and myo-inositol. After SHD treatment, levels of a total of 12 of these 55 different endogenous metabolites were restored in MCAO/R rats. Of these 12 metabolites, 7 were in the brain and 5 in the serum. The 7 metabolites in the brain are 2-piperidinecarboxylic acid, urea, glycine, L-proline, gluconic acid, butyric acid and phosphoric acid (**Figure 6**). The five metabolites in the serum were acetic acid, DL-ornithine, L-ornithine, D-allose and myo-inositol (**Figure 7**).

**Table 1:** Statistical analysis results of identified metabolite changes in the brain.

NO.	Name	Molecular Formula	P value	VIP	M/Z	RT	Fold Change	HMDB
1	Acetic acid	C8H20O3Si2	0.044	1.407	146.9	5.161	0.80	HMDB0000042
2	l-Alanine	C9H23NO2Si2	0.045	1.444	243.9	5.377	0.81	HMDB0000161
3	Propanedioic acid	C9H20O4Si2	0.019	1.604	151.9	6.036	0.69	HMDB0000691
4	Phosphoric acid	C7H21O4PSi2	0.017	1.885	261	6.469	0.32	HMDB0002142
5	L-Valine	C11H27NO2Si2	0.033	1.625	144	6.936	1.39	HMDB0000883
6	Urea	C7H20N2OSi2	0.024	1.674	189	7.475	0.55	HMDB0000294
7	L-Isoleucine	C12H29NO2Si2	0.034	1.628	158	7.955	1.38	HMDB0000172
8	2-Butenedioic acid	C10H20O4Si2	0.001	2.362	43.1	8.669	1.72	HMDB0000176
9	Gluconic acid	C16H35NO6Si3	0.002	2.178	155.9	10.635	0.50	HMDB0000625
10	Propanoic acid	C13H33NO2Si3	0.026	1.468	146.95	10.990	0.76	HMDB0000237
11	Butanoic acid	C13H33NO2Si3	0.015	1.922	174.1	11.021	0.76	HMDB0000039
12	3-Iodo-L-tyrosine	C18H34INO3Si3	0.018	1.691	176.9	12.315	1.41	HMDB0000021
13	L-Asparagine	C13H32N2O3Si3	0.032	1.649	257.9	12.841	0.71	HMDB0000168
14	N-Acetyl-D-glucosamine	C21H50N2O6Si4	0.007	1.946	97	13.463	0.82	HMDB0000215
15	2-Propanone	C15H40NO6PSi4	0.019	1.762	114	14.532	0.67	HMDB0001659
16	L-(-)-Sorbose	C22H55NO6Si5	0.044	1.401	73	15.872	1.28	HMDB0001266
17	D-(-)-Fructose	C22H55NO6Si5	0.026	1.777	308	15.952	1.40	HMDB0000660
18	D-Allose	C22H55NO6Si5	0.022	1.880	146.9	16.105	1.86	HMDB0001151
19	D-Galactose	C22H55NO6Si5	0.003	2.107	73	16.290	1.54	HMDB0000143
20	D-Glucose	C22H55NO6Si5	0.038	1.707	103	16.295	3.33	HMDB0000122
21	Inositol	C24H60O6Si6	0.022	1.678	190.9	19.469	1.31	HMDB0000211
22	Myo-inositol	C24H60O6Si6	0.001	2.060	73	19.481	0.85	HMDB0000211
23	D-(+)-Galactose	C24H61NO6Si6	0.043	1.813	146.9	20.252	1.69	HMDB0000143
24	Heptadecanoic acid	C20H42O2Si	0.029	1.708	73	20.491	0.91	HMDB0002259
25	Octadecanoic acid	C21H44O2Si	0.005	1.834	127.9	22.050	0.67	HMDB0000827
26	11-Eicosenoic acid	C23H46O2Si	0.014	1.619	83	23.876	0.74	HMDB0034296
27	3-Indoleacrylic acid	C23H37NO2Si2	0.050	1.565	173.9	24.224	0.70	HMDB0000734
28	D-Altro-2-Heptulose	C29H74NO10PSi7	0.002	2.147	285.9	24.827	1.57	HMDB0003219

**Table 2:** Statistical analysis results of identified metabolite changes in the serum.

NO.	Name	Molecular Formula	P value	VIP	M/Z	RT	Fold Change	HMDB
1	Glycine	C9H23NO2Si2	0.028	1.59968	173.9	7.414	1.27	HMDB0000123
2	D-(-)-Lactic acid	C9H22O3Si2	0.013	1.67382	73	7.642	0.90	HMDB0001311
3	Propanoic acid	C9H22O3Si2	0.015	1.6416	74.1	7.667	1.19	HMDB0000237
4	L-(+)-Lactic acid	C9H22O3Si2	0.013	1.64365	147	7.675	1.16	HMDB0000190
5	Butanoic acid	C10H24O3Si2	0.010	1.70604	131	9.261	1.78	HMDB0000039
6	1H-Indole-3-ethanamine	C16H28N2Si2	0.001	1.89296	175	12.895	1.87	HMDB0000303
7	Benzaldehyde	C8H7FO3	0.014	1.46248	169.9	13.148	1.30	HMDB0006115
8	L-Isoleucine	C12H29NO2Si2	0.002	1.85282	159	13.585	1.56	HMDB0000172
9	L-Norleucine	C12H29NO2Si2	0.003	1.82305	158	13.587	1.53	HMDB0001645
10	L-Proline	C11H25NO2Si2	0.030	1.30067	142	13.702	1.46	HMDB0000162
11	L-Homoserine	C13H33NO3Si3	0.040	1.41885	73	15.945	1.28	HMDB0000719
12	(R*,S*)-2,3-Dihydroxybutanoic acid	C13H32O4Si3	0.034	1.41502	291	15.948	1.40	HMDB0000498
13	Butanedioic acid	C13H30O5Si3	0.000	2.01385	147.9	18.494	1.88	HMDB0000254
14	2-Butenedioic acid	C10H20O4Si2	0.017	1.49511	73	18.500	1.44	HMDB0000176
15	2-Piperidinecarboxylic acid	C12H27NO2Si2	0.026	1.45946	219	19.226	1.54	HMDB0000070
16	2-Pentanone	C8H18OSi	0.016	1.53469	146.9	20.022	1.61	HMDB0034235
17	Phosphoric acid	C9H22NO4PSSi2	0.007	1.62415	188	21.077	2.35	HMDB0002142
18	Glutamic acid	C14H33NO4Si3	0.014	1.56212	246	21.595	1.61	HMDB0000148
19	1H-Indole-2,3-dione	C17H25NO2Si	0.009	1.63988	73	21.598	1.57	HMDB0061933
20	Pentadecane	C21H44	0.031	1.22446	174	24.455	1.87	HMDB0059886
21	DL-Ornithine	C17H44N2O2Si4	0.046	1.09941	73	25.832	1.51	HMDB32455
22	L-Ornithine	C17H44N2O2Si4	0.012	1.40929	146.9	25.846	1.59	HMDB0000214
23	1,5-Anhydro-D-sorbitol	C18H44O5Si4	0.005	1.72214	75	26.527	1.63	HMDB0002712
24	D-Fructose	C22H55NO6Si5	0.013	1.71509	73	26.913	1.51	HMDB0000660
25	L-(-)-Sorbose	C22H55NO6Si5	0.016	1.709	335.1	26.915	1.70	HMDB0001266
26	D-Allose	C24H61NO6Si6	0.005	1.56378	205	27.434	0.84	HMDB0001151
27	D-Mannose	C24H61NO6Si6	0.034	1.27105	319.1	27.463	0.92	HMDB0000169
28	D-Glucose	C24H61NO6Si6	0.027	1.4162	208	27.467	1.15	HMDB0000122
29	L-Tyrosine	C18H35NO3Si3	0.003	1.63524	218	28.271	1.85	HMDB0000158
30	Myo-Inositol	C24H60O6Si6	0.009	1.36863	305	28.539	2.45	HMDB0000211
31	L-Tryptophan	C20H36N2O2Si3	0.021	1.44616	45.1	33.277	1.60	HMDB0000929
32	D-Glucuronic acid	C21H50O7Si5	0.013	1.46307	217	34.439	2.44	HMDB0000127
33	D-Ribose	C17H42O5Si4	0.050	1.37958	331.15	34.465	2.43	HMDB0000283
34	Probucol	C31H48O2S2	0.015	1.66934	279	44.309	0.19	HMDB0015537

#### 3.4.3 Verification of metabolic pathway analysis and relevant targets

**Figure 8:**
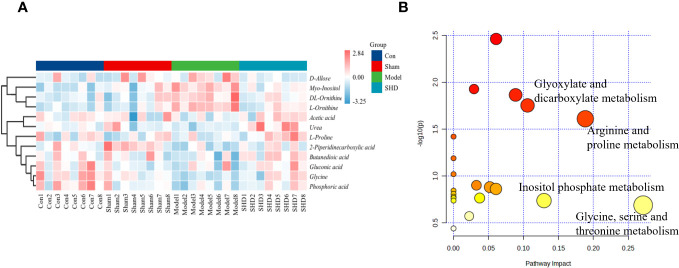
Analysis of differential metabolites in brain and serum in MCAO/R rats after SHD intervention. **(A)** Heat map of metabolite content in serum and brain samples of four groups. **(B)** Pathway analysis of SHD intervention. The metabolic pathways involved in the protection effects of SHD on MCAO/R rats.

**Figure 9:**
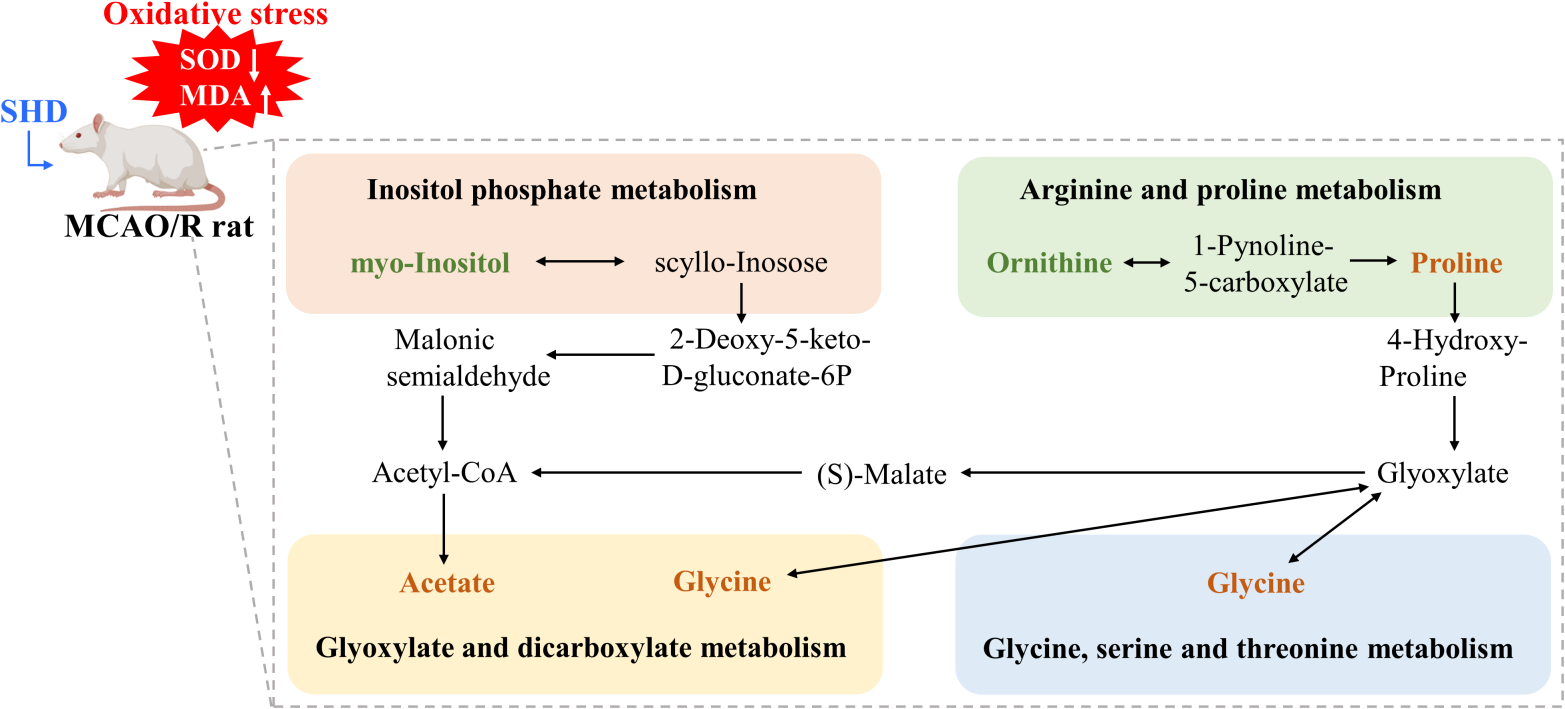
Schematic diagram of the metabolic pathways. Orange and green represent the increase and decrease of d metabolite levels after SHD intervention, respectively.

The cluster analysis of metabolites in the brain and serum is shown as a heat map in **Figure 8A**. Metabolic pathway analysis was conducted on four groups of differential metabolites, to explore correlations between metabolites and major metabolic pathways, with the MetaboAnalyst 5.0 online database. Based on statistical analysis, metabolic pathways with *p* < 0.05 and impact > 0.10 were the major metabolic pathways. The results of metabolic pathway analysis revealed common pathways between the Model and SHD groups, suggesting that SHD acts primarily by affecting these 4 metabolic pathways in MCAO/R rats: glyoxylate and dicarboxylate metabolism, arginine and proline metabolism, inositol phosphate metabolism, and glycine, serine and threonine metabolism (**Figure 8B**). In the KEGG database, 4 major metabolic pathways were linked into a complete metabolic network, elucidating the key metabolic pathways through which SHDs exert their therapeutic effects. (**Figure 9**).

#### 3.4.4 SHD improves disease-related metabolites levels

**Figure 10:**
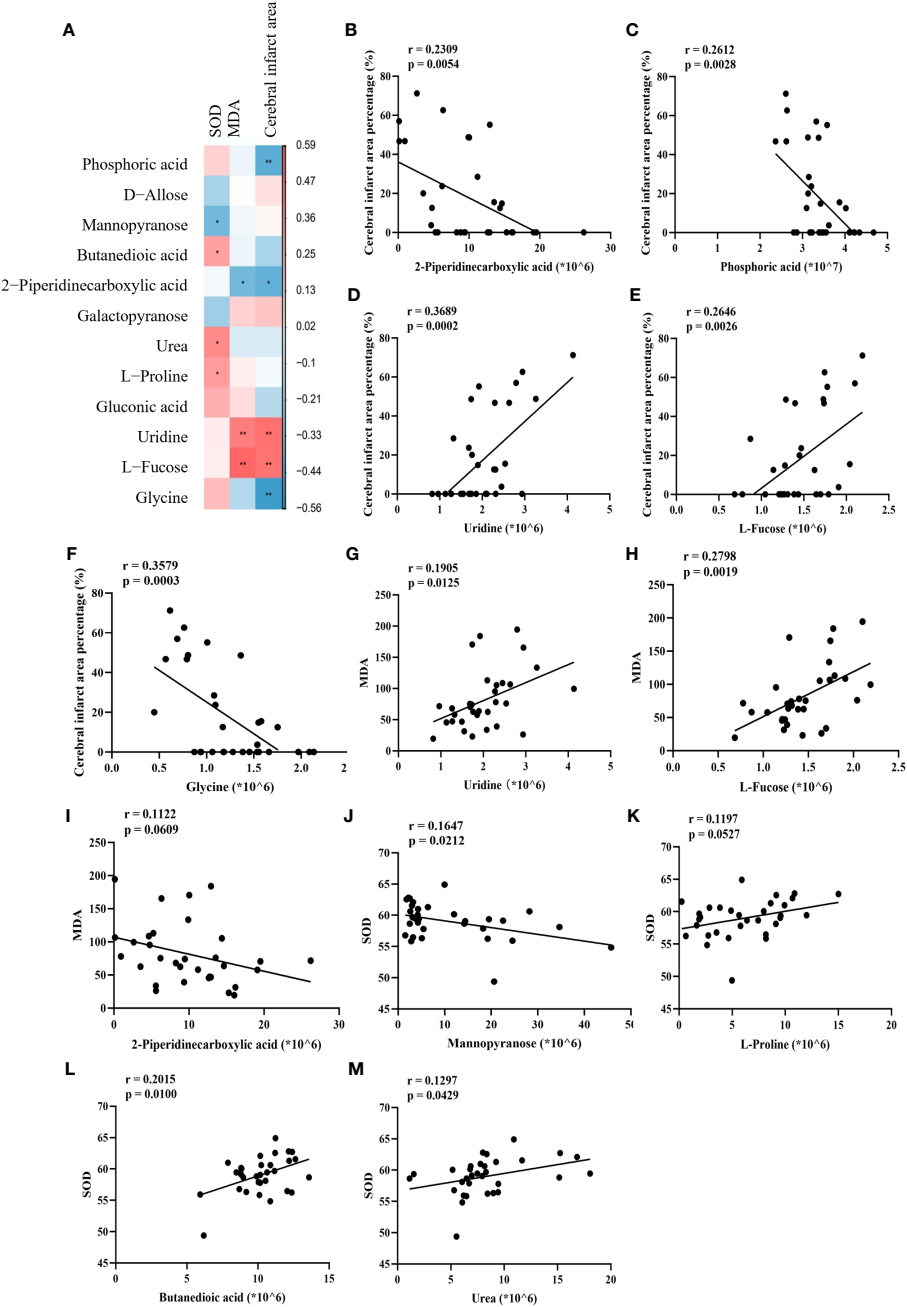
Correlation of disease-related metabolites with disease markers. **(A)** Heat map of Spearman correlation between cerebral infarct area, SOD, MDA and metabolites. **(B–M)** Spearman correlation analysis between percentage of cerebral infarcted area, MDA and SOD with phosphoric acid, mannopyranose, butanedioic acid, 2-piperidinecarboxylic acid, urea, L-proline, uridine, L(-)-fucose and glycine.

To elucidate the relationship between alterations in these 12 associated metabolites and IS, we performed Spearman correlation analyses, plotted correlation heat maps (**Figure 10A**), and screened 10 metabolites that were significantly associated with cerebral infarct area, MDA, and SOD. The correlations for the other 2 metabolites (**Figures 10I, K**) were not significant. The results showed that brain 2-piperidinecarboxylic acid (**Figure 10B**), brain phosphoric acid (**Figure 10C**), and brain glycine (**Figure 10F**) were negatively correlated with cerebral infarct area, and serum uridine (**Figure 10D**) and serum L-fucose (**Figure 10E**) were positively correlated with cerebral infarct area. Serum uridine (**Figure 10G**) and serum L-fucose (**Figure 10H**) were negatively correlated with MDA levels. Serum mannopyranose (**Figure 10J**) was negatively correlated with SOD, and brain butanedioic acid (**Figure 10L**) and brain urea (**Figure 10M**) were positively correlated with SOD. This suggests that SHD may ameliorate ischemic necrosis of brain tissue in tMCAO rats by affecting the levels of disease-related metabolites.

## 4 Discussion

Stroke is the second leading cause of death worldwide. In stroke, the blood supply to certain areas of the brain is interrupted or blocked, preventing the delivery of adequate nutrients and oxygen to brain tissue and ultimately leading to neuronal damage. IS due to embolism is often associated with atrial fibrillation and severe neurological syndromes (25). The incidence of IS is increasing, with high mortality and disability rates. Improving early diagnosis and intervention in IS and developing new therapeutic strategies is now a top priority (26). Vascular interventions and thrombolytic therapy remain the most desirable options for the treatment of IS, but only in certain stroke patients and with very low cure rates and associated with severe adverse effects (27). In almost all patients with IS, secondary ischemia-reperfusion (IR) injury after revascularization is an inevitable consequence (28, 29). IR is the most important cause of cellular necrosis in infarcted lesions, which is further exacerbated by vascular injury or even vascular occlusion (30).

SHD is a well-known classic traditional Chinese medicine prescription for IS. SHD is still used consistently and extensively in modern society for stroke treatment. However, the mechanism of treatment of acute IS by SHD remains uncertain. To confirm the protective effect of SHD, we analyzed cerebral infarct areas in MCAO/R rats. All results indicated that SHD improved the neurological damage and cerebral infarct area in rats. Examination of biochemical indicators showed a significant increase in MDA after MCAO/R operation and in the period of ischemia. It indicated that free radicals and reactive oxygen species cause severe oxidative damage. Meanwhile, SOD may be affected by the ischemia-reperfusion state of the brain due to the imbalance of the antioxidant system. After SHD treatment, the levels of SOD and MDA were restored to alleviate the oxidative stress damage. Stress caused by redox imbalance is an influential factor in ischemia-reperfusion injury and is the initial marker of brain damage. In IR-induced long-term injury, neuroinflammation causes systemic inflammation, which further leads to progressive dysfunction of peripheral organs (31). The results of brain pathology sections indicated that SHD had a ameliorative effect on brain damage after IR by decreasing neuronal necrosis and improving neuronal cell structure and inflammatory cell infiltration. At the same time, no significant side effects of SHD on other organs were observed, suggesting that SHD is safe at the doses at which it exerts its therapeutic effects.

In recent years, a growing number of studies have identified the same signature metabolites from patients with cerebral ischemia and animal models that improve the diagnosis and predict the outcome of IS (32). Gender is an important variable in the prevalence of stroke. There are 55,000 more women than men who suffer a stroke each year. Women bear a greater risk of disease than men (33). The aim of this study was to determine the therapeutic effect of SHD on ischemic stroke. To exclude female-unique factors such as sex hormones, exogenous estrogens, and pregnancy, we performed a GC-MS based metabolomics study using male rats. Brain and serum metabolite analyses were utilized to assess the therapeutic effects and potential mechanisms of SHD in MCAO/R rats.

We identified 55 differential metabolites between the Sham and Model groups. These 55 altered metabolites are potential biomarkers of IS, which will facilitate later monitoring of disease progression in the clinic. After SHD intervention, the levels of 7 and 5 differential metabolites were restored in brain and serum, respectively. Of these 12 differential metabolites that were restored, changes in 8 were significantly different. The Model group had decreased levels of urea, glycine, L-proline, gluconic acid and butanedioic acid, and increased levels of myo-inositol, L-ornithine, and DL-ornithine compared with the Sham group. After treatment with SHD, the levels of the above metabolites were restored. These 12 metabolites were mainly related to 4 metabolic pathways, including glyoxylate and dicarboxylate metabolism, arginine and proline metabolism, inositol phosphate metabolism, and glycine, serine and threonine metabolism. Many metabolic problems have been shown to lead to stroke or stroke-like episodes (34).

Glyoxalate and dicarboxylic acid metabolism is closely correlated with the TCA cycle. The chain cycle of oxidative decarboxylation of glyoxylate serves as a native metabolic cycle analog of the TCA cycle. In the TCA cycle, glyoxylate can be converted to carbon dioxide and produce aspartate (35). Gluconic acid is associated with oxidative stress and is a predictor of hyperglycemia and cytotoxic brain injury after stroke (36). Arginine and proline metabolism have been shown to be associated with axon regeneration, and arginine is a necessary raw material for axon regeneration (37). L-ornithine produced during its metabolism has recently been shown to be one of the biomarkers used for early detection of stroke (38). L-ornithine transcarbamylase deficiency leads to elevated ornithine levels, which in turn cause impaired urea cycling that can lead to stroke-like episodes (39), impaired mitochondrial function and reduced antioxidant capacity (40). After IS, plasma L-proline levels are reduced (41). Excitotoxicity mediated by N-methyl-D-aspartate receptors (NMDARs) regulated by L-proline, and is one of the main reasons of neuronal death after stroke (42, 43). It has been shown that neuroprotection and neurorepair can be promoted in acute ischemic stroke models by inhibiting L-proline uptake in the brain (43). Glycine can regulate microglial polarization after IS and indirectly inhibit ischemia-induced neuronal death and functional recovery (44). Through the miR-19a-3p/AMPK/GSK-3β/HO-1 pathway, glycine ameliorates apoptosis, inflammatory response and dysregulated glucose metabolism in IS (45). In addition, *in vitro* experiments showed that low doses of glycine improved NMDAR function, but high doses of glycine induced NMDAR internalization and thus neuroprotective effects in IS (46). Inositol phosphate metabolism has been shown to be abnormal in a rat stroke model (47). Inositol and phosphatidylinositol reduce insulin resistance, improve insulin sensitivity, and have unique roles in energy metabolism and metabolic disorders (48). Phosphatidylinositol 3-kinase is a phosphorylation product of inositol phospholipids and is involved in the immune response. Electrical stimulation of the cerebral cortex exerts anti-apoptotic, angiogenic and anti-inflammatory effects via the phosphatidylinositol 3-kinase/Akt pathway in rats with IS (49). Taken together, amino acid metabolism and carbohydrate metabolism are closely related to IS. The present study showed that the differences of metabolite between the Model group and the Sham group had improved to some extent after SHD intervention, and some metabolite levels were restored. This suggested that therapeutic effect of SHD on IR-induced metabolic disorders.

## 5 Conclusion

In this research, we integrated pharmacodynamic and metabolomic approaches to investigate firstly the therapeutic effects of SHD on the MCAO/R rat model and its underlying mechanisms. Our findings indicated that SHD treatment ameliorated MCAO/R-induced IS symptoms, ameliorated oxidative stress, attenuated brain damage and had a protective effect on damaged nerves. Brain and serum metabolomic analyses suggested that SHD treatment could significantly affect 4 metabolic pathways. The neuroprotective effects of SHD were primarily mediated through modulation of amino acid metabolism and carbohydrate metabolism, and nine metabolites that were altered after SHD treatment were significantly associated with disease. In conclusion, these findings deepen the understanding of the mechanisms by which SHD treats IS and suggests that SHD is promising drug candidate for intervening in the developmental process of IS.

## Data availability statement

The original contributions presented in the study are included in the article/**Supplementary Material**. Further inquiries can be directed to the corresponding authors.

## Ethics statement

The animal study was approved by China Academy of Chinese Medical Sciences. The study was conducted in accordance with the local legislation and institutional requirements.

## Author contributions

RL: Writing – original draft. SC: Writing – original draft. YC: Writing – review & editing. MZ: Conceptualization, Writing – review & editing. XG: Data curation, Writing – review & editing. YH: Writing – review & editing.

## References

[1] Hankey GJ. Stroke. Lancet (2017) 389:641–54. doi: 10.1016/S0140-6736(16)30962-X 27637676

[2] Wang X-G Zhu D-D Li N Huang Y-L Wang Y-Z Zhang T. Scorpion venom heat-resistant peptide is neuroprotective against cerebral ischemia-reperfusion injury in association with the NMDA-MAPK pathway. Neurosci Bull (2020) 36:243–53. doi: 10.1007/s12264-019-00425-1 PMC7056763 31502213

[3] Pénzes M Túrós D Máthé D Szigeti K Hegedűs N Rauscher AÁ. Direct myosin-2 inhibition enhances cerebral perfusion resulting in functional improvement after ischemic stroke. Theranostics (2020) 10:5341–56. doi: 10.7150/thno.42077 PMC7196296 32373216

[4] Catanese L Tarsia J Fisher M. Acute ischemic stroke therapy overview. Circ Res (2017) 120:541–58. doi: 10.1161/CIRCRESAHA.116.309278 28154103

[5] IST-3 collaborative group Sandercock P Wardlaw JM Lindley R Dennis M Cohen G. The benefits and harms of intravenous thrombolysis with recombinant tissue plasminogen activator within 6 h of acute ischaemic stroke (the third international stroke trial [IST-3]): a randomised controlled trial. Lancet (2012) 379:2352–63. doi: 10.1016/S0140-6736(12)60768-5 PMC3386495 22632908

[6] Seto S-W Chang D Jenkins A Bensoussan A Kiat H. Angiogenesis in ischemic stroke and angiogenic effects of Chinese herbal medicine. J Clin Med (2016) 5:56. doi: 10.3390/jcm5060056 27275837 PMC4929411

[7] Wang M Yao M Liu J Takagi N Yang B Zhang M. Ligusticum chuanxiong exerts neuroprotection by promoting adult neurogenesis and inhibiting inflammation in the hippocampus of ME cerebral ischemia rats. J Ethnopharmacology (2020) 249:112385. doi: 10.1016/j.jep.2019.112385 31730888

[8] Zhou X Seto SW Chang D Kiat H Razmovski-Naumovski V Chan K. Synergistic effects of chinese herbal medicine: A comprehensive review of methodology and current research. Front Pharmacol (2016) 7:201. doi: 10.3389/fphar.2016.00201 27462269 PMC4940614

[9] Liu N Liu C Yang Y Ma G Wei G Liu S. Xiao-Xu-Ming decoction prevented hemorrhagic transformation induced by acute hyperglycemia through inhibiting AGE-RAGE-mediated neuroinflammation. Pharmacol Res (2021) 169:105650. doi: 10.1016/j.phrs.2021.105650 33964468

[10] Zhang S Kong D Ma G Liu C Yang Y Liu S. Long-term administration of salvianolic acid A promotes endogenous neurogenesis in ischemic stroke rats through activating Wnt3a/GSK3β/β-catenin signaling pathway. Acta Pharmacol Sin (2022) 43:2212–25. doi: 10.1038/s41401-021-00844-9 PMC9433393 35217812

[11] Li Y Liang W Guo C Chen X Huang Y Wang H. Renshen Shouwu extract enhances neurogenesis and angiogenesis via inhibition of TLR4/NF-κB/NLRP3 signaling pathway following ischemic stroke in rats. J Ethnopharmacology (2020) 253:112616. doi: 10.1016/j.jep.2020.112616 32007631

[12] Yang L Su X Lu F Zong R Ding S Liu J. Serum and brain metabolomic study reveals the protective effects of Bai-Mi-Decoction on rats with ischemic stroke. Front Pharmacol (2022) 13:1005301. doi: 10.3389/fphar.2022.1005301 36506507 PMC9729534

[13] Ye Y Zhu Y Xin X Zhang J Zhang H Li D. Efficacy of Chinese herbal medicine for tPA thrombolysis in experimental stroke: A systematic review and meta-analysis. Phytomedicine (2022) 100:154072. doi: 10.1016/j.phymed.2022.154072 35349833

[14] Fu D-L Li J-H Shi Y-H Zhang X-L Lin Y Zheng G-Q. Sanhua decoction, a classic herbal prescription, exerts neuroprotection through regulating phosphorylated tau level and promoting adult endogenous neurogenesis after cerebral ischemia/reperfusion injury. Front Physiol (2020) 11:57. doi: 10.3389/fphys.2020.00057 32116767 PMC7026024

[15] Zheng L Meng L Liang H Yang J. Sanhua decoction: Current understanding of a traditional herbal recipe for stroke. Front Neurosci (2023) 17:1149833. doi: 10.3389/fnins.2023.1149833 37123364 PMC10133510

[16] Liu T Zhou J Cui H Li P Luo J Li T. iTRAQ-based quantitative proteomics reveals the neuroprotection of rhubarb in experimental intracerebral hemorrhage. J Ethnopharmacology (2019) 232:244–54. doi: 10.1016/j.jep.2018.11.032 30502478

[17] Li X Chu S Liu Y Chen N. Neuroprotective effects of anthraquinones from rhubarb in central nervous system diseases. Evidence-Based Complementary Altern Med (2019) 2019:e3790728. doi: 10.1155/2019/3790728 PMC6541978 31223328

[18] Ruan Y Jin X Ji H Zhu C Yang Y Zhou Y. Water extract of Notopterygium incisum alleviates cold allodynia in neuropathic pain by regulation of TRPA1. J Ethnopharmacology (2023) 305:116065. doi: 10.1016/j.jep.2022.116065 36587876

[19] Zhu S Liu F Zhang R Xiong Z Zhang Q Hao L. Neuroprotective potency of neolignans in magnolia officinalis cortex against brain disorders. Front Pharmacol (2022) 13:857449. doi: 10.3389/fphar.2022.857449 35784755 PMC9244706

[20] Liu X Chen X Zhu Y Wang K Wang Y. Effect of magnolol on cerebral injury and blood brain barrier dysfunction induced by ischemia-reperfusion in *vivo* and in *vitro* . Metab Brain Dis (2017) 32:1109–18. doi: 10.1007/s11011-017-0004-6 28378105

[21] Choi B-K Kim T-W Lee D-R Jung W-H Lim J-H Jung J-Y. A polymethoxy flavonoids-rich Citrus aurantium extract ameliorates ethanol-induced liver injury through modulation of AMPK and Nrf2-related signals in a binge drinking mouse model. Phytotherapy Res (2015) 29:1577–84. doi: 10.1002/ptr.5415 26178909

[22] Lu Y Huang Y Sun M Li W. Determination of active ingredients and prediction of targetin Sanhua Decoction for cerebral ischemia based on HPLC and network pharmacology methods. Int J Traditional Chin Med (2021) 43:1109–15. doi: 10.3760/cma.j.cn115398-20210413-00140

[23] YingHuang U Gao S Gong Z Li W Xiao-junGou U Sun J. Mechanism of sanhua decoction in the treatment of ischemic stroke based on network pharmacology methods and experimental verification. BioMed Res Int (2022) 2022:e7759402. doi: 10.1155/2022/7759402 PMC8799339 35097126

[24] Benedek A Móricz K Jurányi Z Gigler G Lévay G Hársing LG. Use of TTC staining for the evaluation of tissue injury in the early phases of reperfusion after focal cerebral ischemia in rats. Brain Res (2006) 1116:159–65. doi: 10.1016/j.brainres.2006.07.123 16952339

[25] Hart RG. Atrial fibrillation and prevention of embolic stroke. Stroke (2021) 52:e55–7. doi: 10.1161/STROKEAHA.120.030420 33423514

[26] Mao R Zong N Hu Y Chen Y Xu Y. Neuronal death mechanisms and therapeutic strategy in ischemic stroke. Neurosci Bull (2022) 38:1229–47. doi: 10.1007/s12264-022-00859-0 PMC9554175 35513682

[27] Pirson FAV Boodt N Brouwer J Bruggeman AAE den Hartog SJ Goldhoorn R-JB. Endovascular treatment for posterior circulation stroke in routine clinical practice: results of the multicenter randomized clinical trial of endovascular treatment for acute ischemic stroke in the Netherlands registry. Stroke (2022) 53:758–68. doi: 10.1161/STROKEAHA.121.034786 34753304

[28] Villringer K Cuesta BES Ostwaldt A-C Grittner U Brunecker P Khalil AA. DCE-MRI blood–brain barrier assessment in acute ischemic stroke. Neurology (2017) 88:433–40. doi: 10.1212/WNL.0000000000003566 28031392

[29] Arba F Piccardi B Palumbo V Biagini S Galmozzi F Iovene V. Blood–brain barrier leakage and hemorrhagic transformation: The Reperfusion Injury in Ischemic StroKe (RISK) study. Eur J Neurol (2021) 28:3147–54. doi: 10.1111/ene.14985 34143500

[30] Jayaraj RL Azimullah S Beiram R Jalal FY Rosenberg GA. Neuroinflammation: friend and foe for ischemic stroke. J Neuroinflamm (2019) 16:142. doi: 10.1186/s12974-019-1516-2 PMC6617684 31291966

[31] DeLong JH Ohashi SN O’Connor KC Sansing LH. Inflammatory responses after ischemic stroke. Semin Immunopathol (2022) 44:625–48. doi: 10.1007/s00281-022-00943-7 35767089

[32] Zhang R Meng J Wang X Pu L Zhao T Huang Y. Metabolomics of ischemic stroke: insights into risk prediction and mechanisms. Metab Brain Dis (2022) 37:2163–80. doi: 10.1007/s11011-022-01011-7 35612695

[33] Demel SL Kittner S Ley SH McDermott M Rexrode KM. Stroke risk factors unique to women. Stroke (2018) 49:518–23. doi: 10.1161/STROKEAHA.117.018415 PMC5909714 29438077

[34] Mastrangelo M Ricciardi G Giordo L Michele MD Toni D Leuzzi V. Stroke and stroke-like episodes in inborn errors of metabolism: Pathophysiological and clinical implications. Mol Genet Metab (2022) 135:3–14. doi: 10.1016/j.ymgme.2021.12.014 34996714

[35] Springsteen G Yerabolu JR Nelson J Rhea CJ Krishnamurthy R. Linked cycles of oxidative decarboxylation of glyoxylate as protometabolic analogs of the citric acid cycle. Nat Commun (2018) 9:91. doi: 10.1038/s41467-017-02591-0 29311556 PMC5758577

[36] Ament Z Bevers MB Wolcott Z Kimberly WT Acharjee A. Uric acid and gluconic acid as predictors of hyperglycemia and cytotoxic injury after stroke. Transl Stroke Res (2021) 12:293–302. doi: 10.1007/s12975-020-00862-5 33067777 PMC7933067

[37] Zhang J Jiang C Liu X Jiang C Cao Q Yu B. The metabolomic profiling identifies N, N-dimethylglycine as a facilitator of dorsal root ganglia neuron axon regeneration after injury. doi: 10.1096/fj.202101698R 35394692

[38] Tao S Xiao X Li X Na F Na G Wang S. Targeted metabolomics reveals serum changes of amino acids in mild to moderate ischemic stroke and stroke mimics. Front Neurol (2023) 14:1153193. doi: 10.3389/fneur.2023.1153193 37122289 PMC10140586

[39] Gowda VK Gupta P Shivappa SK Benakappa N. Recurrent stroke like episodes secondary to ornithine transcarbamylase deficiency. Indian J Pediatr (2020) 87:852–3. doi: 10.1007/s12098-020-03193-3 32008222

[40] Zanatta Â Rodrigues MDN Amaral AU Souza DG Quincozes-Santos A Wajner M. Ornithine and homocitrulline impair mitochondrial function, decrease antioxidant defenses and induce cell death in menadione-stressed rat cortical astrocytes: potential mechanisms of neurological dysfunction in HHH syndrome. Neurochem Res (2016) 41:2190–8. doi: 10.1007/s11064-016-1933-x 27161368

[41] Fan D Krishnamurthi R Harris P Barber PA Guan J. Plasma cyclic glycine proline/IGF-1 ratio predicts clinical outcome and recovery in stroke patients. Ann Clin Trans Neurol (2019) 6:669–77. doi: 10.1002/acn3.743 PMC6469247 31019991

[42] Goulart VAM Sena MM Mendes TO Menezes HC Cardeal ZL Paiva MJN. Amino acid biosignature in plasma among ischemic stroke subtypes. BioMed Res Int (2019) 2019:e8480468. doi: 10.1155/2019/8480468 PMC6360633 30800679

[43] Carvalho GA Chiareli RA Marques BL Parreira RC de Souza Gil E de Carvalho FS. L-proline transporter inhibitor (LQFM215) promotes neuroprotection in ischemic stroke. Pharmacol Rep (2023) 75:276–92. doi: 10.1007/s43440-023-00451-x 36719635

[44] Liu R Liao X-Y Pan M-X Tang J-C Chen S-F Zhang Y. Glycine Exhibits Neuroprotective Effects in Ischemic Stroke in Rats through the Inhibition of M1 Microglial Polarization via the NF-κB p65/Hif-1α Signaling Pathway. J Immunol (2019) 202:1704–14. doi: 10.4049/jimmunol.1801166 30710045

[45] Chen Z-J Zhao X-S Fan T-P Qi H-X Li D. Glycine improves ischemic stroke through miR-19a-3p/AMPK/GSK-3&beta;/HO-1 pathway. DDDT (2020) 14:2021–31. doi: 10.2147/DDDT.S248104 PMC7260540 32546967

[46] Cappelli J Khacho P Wang B Sokolovski A Bakkar W Raymond S. Glycine-induced NMDA receptor internalization provides neuroprotection and preserves vasculature following ischemic stroke. iScience (2022) 25:103539. doi: 10.1016/j.isci.2021.103539 34977503 PMC8689229

[47] Lin TN Liu TH Xu J Hsu CY Sun GY. Brain polyphosphoinositide metabolism during focal ischemia in rat cortex. Stroke (1991) 22:495–8. doi: 10.1161/01.STR.22.4.495 1850877

[48] Chatree S Thongmaen N Tantivejkul K Sitticharoon C Vucenik I. Role of inositols and inositol phosphates in energy metabolism. Molecules (2020) 25:5079. doi: 10.3390/molecules25215079 33139672 PMC7663797

[49] Baba T Kameda M Yasuhara T Morimoto T Kondo A Shingo T. Electrical stimulation of the cerebral cortex exerts antiapoptotic, angiogenic, and anti-inflammatory effects in ischemic stroke rats through phosphoinositide 3-kinase/akt signaling pathway. Stroke (2009) 40:e598–605. doi: 10.1161/STROKEAHA.109.563627 19762690

