# Supplementary material for: Brain and serum metabolomic studies reveal therapeutic effects of san hua decoction in rats with ischemic stroke
Source: Front Endocrinol (Lausanne). 2023 Nov 30;14:1289558. doi: 10.3389/fendo.2023.1289558 (PMC10720749; doi:10.3389/fendo.2023.1289558)
Supplement: Supplementary file 1 [file DataSheet_1.docx]

Supplementary Material

Article Title

Ruisi Liu^†^, Shengxuan Cao^†^, Yufeng Cai, Mingmei Zhou*, Xiaojun Gou*, Ying Huang*

*** Correspondence:**

Mingmei Zhou: [zhoumm368@163.com](mailto:zhoumm368@163.com)

Xiaojun Gou: gouxiaojun1975@163.com

Ying Huang: huangying0518@126.com

# Supplementary Figures and Tables

## Supplementary Figures

**
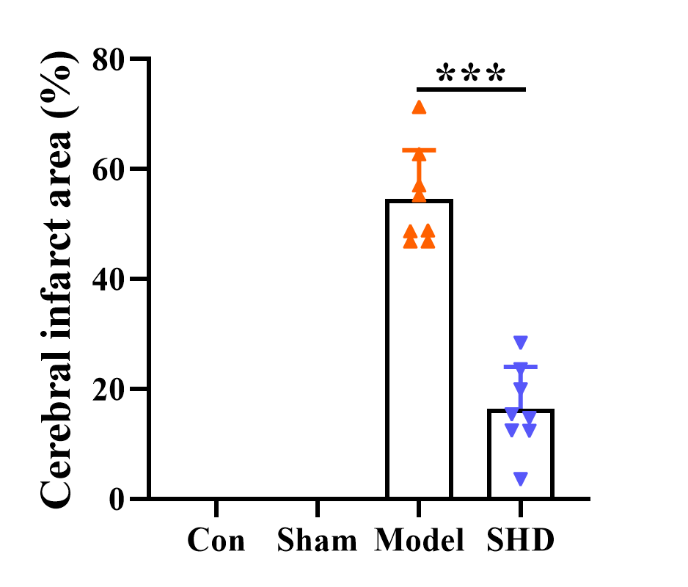
**

**Figure 1.** Cerebral infarct area examination. All values were presented as the mean ± SD. *p < 0.05, **p < 0.01, ***p < 0.001 relative to the Model group.

## Supplementary Tables

**Table 1.** Cerebral infarct area examination. (*$\overline{x}$*±s，n=8)

| Group | Infarct area (%) |
| --- | --- |
| Control | 0.00±0.00 |
| Sham | 0.00±0.00 |
| Model | 54.62±8.78** |
| SHD | 16.41±7.64**## |

Data were expressed as the mean ± SD. ** *p*＜0.01, relative to the Sham group； ## *p*＜0.01, relative to the Model group.

**Table 2.** Effect of SHD on SOD and MDA in serum of rats with cerebral ischemia-reperfusion. (*$\overline{x}$*±s，n=8)

| Group | SOD (%) | MDA (uM) |
| --- | --- | --- |
| Control | 57.54±1.81 | 63.48±25.05** |
| Sham | 59.57±1.29 | 40.20±18.36** |
| Model | 56.98±3.79 | 141.52±43.29## |
| SHD | 61.93±1.78 | 80.60±19.95**## |

Data were expressed as the mean ± SD. ## *p* ＜0. 01, relative to the Sham group; * *p* ＜0. 05，** *p* ＜0. 01, relative to the Model group.
